# Activation of the SARS-CoV-2 receptor *Ace2* by cytokines through pan JAK-STAT enhancers

**DOI:** 10.1101/2020.05.11.089045

**Published:** 2020-05-11

**Authors:** Lothar Hennighausen, Hye Kyung Lee

**Affiliations:** Laboratory of Genetics and Physiology, National Institute of Diabetes and Digestive and Kidney Diseases, US National Institutes of Health, Bethesda, Maryland 20892, USA

## Abstract

ACE2, in concert with the protease TMPRSS2, binds the novel coronavirus SARS-CoV-2 and facilitates its cellular entry. The *ACE2* gene is expressed in SARS-CoV-2 target cells, including Type II Pneumocytes (Ziegler, 2020), and is activated by interferons. Viral RNA was also detected in breast milk (Wu et al., 2020), raising the possibility that *ACE2* expression is under the control of cytokines through the JAK-STAT pathway. Here we show that *Ace2* expression in mammary tissue is induced during pregnancy and lactation, which coincides with the establishment of a candidate enhancer. The prolactin-activated transcription factor STAT5 binds to tandem sites that coincide with activating histone enhancer marks and additional transcription components. The presence of pan JAK-STAT components in mammary alveolar cells and in Type II Pneumocytes combined with the autoregulation of both STAT1 and STAT5 suggests a prominent role of cytokine signaling pathways in cells targeted by SARS-CoV-2.

## Introduction

ACE2, the receptor for SARS-CoV (Imai et al., 2005) and SARS-CoV-2 (Hoffmann et al., 2020), has been identified in several target cells, including absorptive Enterocytes (Lamers et al., 2020), secretory goblet cells (Zhao, 2020), the olfactory system (Brann, 2020) and several epithelial cell types (Brann, 2020; Lukassen et al., 2020; Qi et al., 2020). A study in pneumocytes demonstrated that *ACE2* expression is induced by interferons (Ziegler, 2020), possibly through the transcription factors Signal Transducer and Activator of Transcription (STAT) 1 and 2, as the authors suggest. The STAT family is comprised of seven transcription factors (STAT1, 2, 3, 4, 5A, 5B and 6) that are activated by type I and II cytokines through their respective receptors and the JAK/TYK2 family of tyrosine kinases (Stark and Darnell, 2012). Although each cytokine receptor has some preference for individual STAT members, it has become clear that any given cytokine can activate several, if not all, STAT members, which subsequently bind to a shared DNA motif, the gamma interferon activated sequence (GAS) (Hennighausen and Robinson, 2008). This permits individual genes to be activated by more than one cytokine through different receptors and several STAT family members.

While SARS-CoV-2 infection of lung epithelium is driving the disease, disturbances in other cell types (Qi et al., 2020), such as the olfactory system (Brann, 2020), have been observed. SARS-CoV-2 RNA has also been detected in breast milk of infected patients (Wu et al., 2020) suggesting that the virus can enter differentiated mammary alveolar cells and be vertically transmitted through breast feeding. Based on the overlapping activities of JAK-STAT components and their potential redundancy, it is likely that *ACE2* expression is activated by a wide range of cytokines through STATs 1, 2, 3 and 5. This has profound implications for strategies to mitigate ACE2 levels. Interfering with individual STATs will result in the compensational recruitment of other STAT members to cytokine receptors (Cui et al., 2007) with all its transcriptional consequences (Hennighausen and Robinson, 2008; Shin et al., 2016).

## Results and Discussion

**Figure 1.**
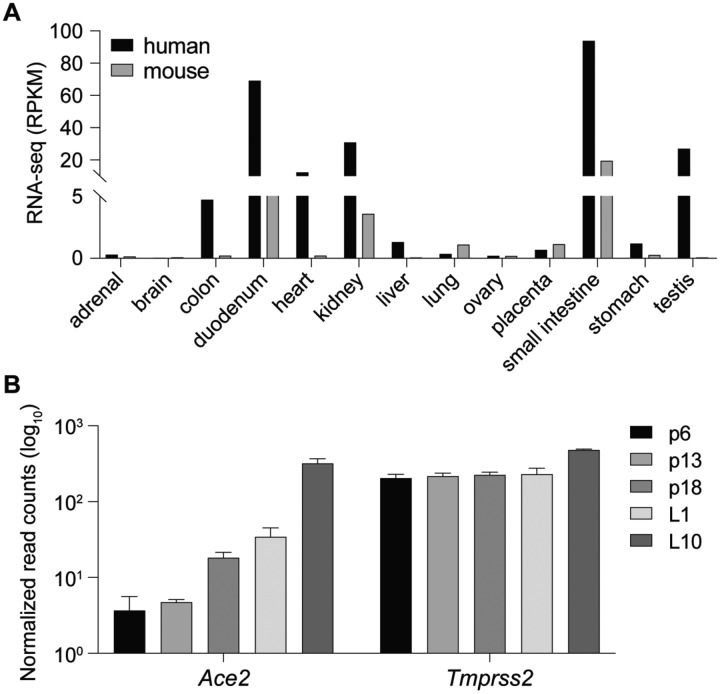
Ace2 is activated during lactation. **(A)** RNA-seq data from ENCODE demonstrate the presence of *ACE2* in several human and mouse tissues. **(B)** *Ace2* and *Tmprss2* mRNA levels in mouse mammary tissue at different stages of pregnancy and lactation were measured by RNA-seq. Day 6 of pregnancy (p6), p13, p18 and days 1 of lactation (L1) and L10.

*Ace2* mRNA levels vary widely between cell types, with high expression detected in lactating mammary and intestinal tissues (Figure 1A–B) and Type II Pneumocytes (Ziegler, 2020). To explore the possibility that *Ace2* gene expression in SARS-CoV-2 target cells is regulated not only by interferons but also by a range of cytokines through the family of STAT transcription factors, we mined available scRNA-seq data (Ziegler, 2020) (Table 1). Interferon receptors (IFNAR) and its downstream mediators JAK1, JAK2, TYK2 as well as STATs 1, 3 and 5 are highly expressed, thus supporting the mechanism of *ACE2* induction by IFN-α/β and IFN-γ. STAT1 levels increase sharply in cells treated with IFNs, supporting the notion of an autoregulatory loop (Yuasa and Hijikata, 2016). Moreover, these expression data point to the presence of functional STAT3 and STAT5 signaling cascades. Interleukin receptors, such as IL-7R, that are dependent on the common gamma chain (IL2RG), JAK1 and JAK3 are also highly expressed.

**Figure 2.**
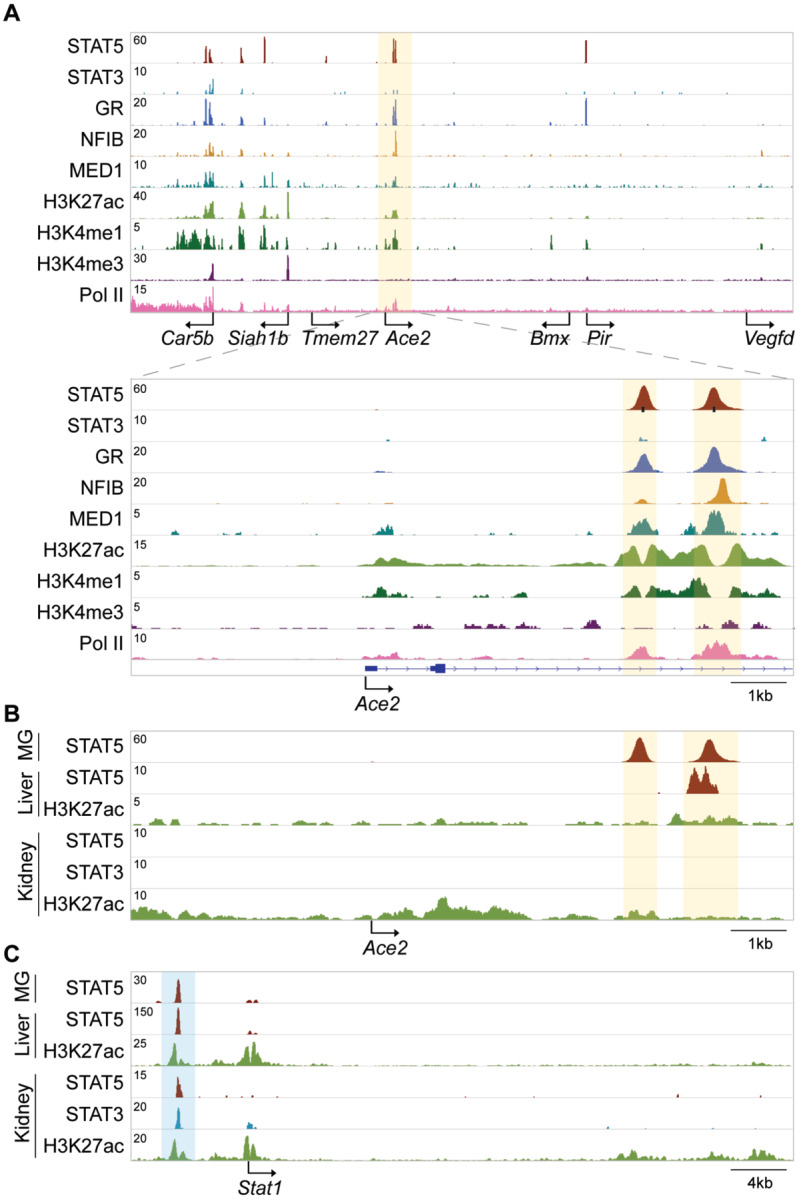
Establishment of a candidate *Ace2* enhancer during lactation. **(A)** ChIP-seq data for STAT5, STAT3, GR, NFIB, MED1 and histone markers H3K27ac and H3K4me3 provided structural information of the locus including the *Ace2* gene in day ten lactating mammary tissue. Solid arrows indicate the orientation of genes. The black bars indicate GAS motifs (STAT binding sites). The orange shades the candidate regulatory elements. **(B-C)** ChIP-seq profiles showed STAT binding at the candidate *Ace2* enhancer and the putative *Stat1* autoregulatory enhancer in mouse liver and kidney. The orange and blue shades indicate putative regulatory elements in each locus.

The presence of a wide range of cytokine receptors, JAKs and STATs, suggests that *Ace2* might be activated by a broad selection of extracellular cues and most cytokines, including growth hormone and prolactin. We have tested this premise and explored whether *Ace2* is activated in mouse mammary tissue through STAT transcription factors. Gene expression in mammary epithelium during pregnancy and lactation is activated by prolactin through STAT5 (Liu et al., 1997). We observed an approximately 100-fold increase of *Ace2* mRNA during pregnancy and lactation (Figure 1B), which coincided with the establishment of a putative enhancer (Figure 2A). *Tmprss2* mRNA levels were similar throughout pregnancy and lactation (Figure 1B), suggesting that its expression is not under overt control of the JAK/STAT pathway. STAT5 was recruited to two distinct GAS (bona fide STAT binding motifs) in the candidate enhancer and co-occupancy of the glucocorticoid receptor (GR), nuclear factor 1 B (NFIB) and mediator complex subunit 1 (MED1) is likely not through their individual recognition motifs but through contacting STAT5. The presence of H3K4me1 enhancer marks, H3K27ac marks and RNA polymerase II (Pol II) occupancy further supports the validity of this regulatory region. Of note, no STAT3 occupancy was observed, suggesting a predominance of STAT5. In contrast to mammary tissue, limited STAT5 binding was observed in liver and no STAT5 and STAT3 binding was observed in kidney tissue (Figure 2B). The putative autoregulatory enhancer in the *Stat1* gene served as a positive control for STAT binding (Figure 2C).

Our study demonstrates the presence of pan JAK/STAT components in Type II Pneumocytes, suggesting that *ACE2* is not only activated by IFN-α/β and IFN-γ but also by other cytokines. Moreover, we demonstrate an ~100-fold increase of *Ace2* expression by pregnancy and lactation hormones in mouse mammary tissue. Future inquiries aimed at understanding the mechanism of *ACE2* gene regulation in potential SARS-CoV-2 target cells need to address the pan JAK-STAT pathway as well as steroid hormones, which might explain some of the sex differences seen in Covid-19 morbidity and mortality. Such investigations would need to include experimental approaches that comprehensively interrogate regulatory elements controlling *ACE2* expression *in vivo* in human tissues, both in males and females at different ages. Since underlying preexisting conditions, such as obesity, diabetes and high blood pressure, can affect the severity and progression of Covid-19, it would prudent to take this into account when analyzing the control of *Ace2* regulation.

## Materials and Methods

### Chromatin immunoprecipitation sequencing (ChIP-seq) analysis.

Quality filtering and alignment of the raw reads was done using Trimmomatic (Bolger et al., 2014) (version 0.36) and Bowtie (Langmead et al., 2009) (version 1.1.2), with the parameter ‘−m 1’ to keep only uniquely mapped reads, using the reference genome mm10. Picard tools (Broad Institute. Picard, http://broadinstitute.github.io/picard/. 2016) was used to remove duplicates and subsequently, Homer (Heinz et al., 2010) (version 4.8.2) and deepTools (Ramirez et al., 2016) (version 3.1.3) software was applied to generate bedGraph files, seperately. Integrative Genomics Viewer (Thorvaldsdottir et al., 2013) (version 2.3.81) was used for visualization. Coverage plots were generated using Homer (Heinz et al., 2010) software with the bedGraph from deepTools as input. R and the packages dplyr (https://CRAN.R-project.org/package=dplyr) and ggplot2 (Love et al., 2014) were used for visualization. Each ChIP-seq experiment was conducted for two replicates. Sequence read numbers were calculated using Samtools (Masella et al., 2016) software with sorted bam files. The correlation between the ChIP-seq replicates was computed using deepTools using Spearman correlation.

### RNA-seq analysis.

RNA-seq reads were analyzed using Trimmomatic (Bolger et al., 2014) (version 0.36) to check read quality (with following parameters: LEADING: 3, TRAILING: 3, SLIDINGWINDOW: 4:20, MINLEN: 36). The alignment was performed in Bowtie aligner (Langmead et al., 2009) (version 1.1.2) using paired end mode.

## Figures and Tables

**Table 1.** mRNA levels of genes associated with the pan JAK-STAT pathway in primary human basal epithelial cells. scRNA-seq data were extracted from the study by Ziegler and colleagues(Ziegler, 2020). The human bronchial cell line (BEAS-2B) and airway basal cells from human donors had been exposed to interferons (IFNa2 and IFNg) and cytokines (IL4 and IL17A). scRNA-seq libraries were generated with 15,000 cells. mRNA levels for genes in JAK/STAT signaling pathway were collected from the data and averages of independent biological replicates were normalized to the value of untreated group. Genes that were regulated more than 3-fold by interferons and cytokines are marked in red and highlighted colors.

Cells	BEAS cell					Human1334					Human2344				
N	12	3	3	3	3	9	3	3	3	3	12	3	3	3	3
drug (10nq/mL)	Untre ated	IFNa2	IFNg	IL4	IL17A	Untre ated	IFNa2	IFNg	IL4	IL17A	Untre ated	IFNa2	IFNg	IL4	IL17A
ACE2	1.00	2.18	6.59	0.00	0.00	1.00	17.53	8.40	0.96	1.04	1.00	5.69	3.42	2.30	0.58
TMPRSS2	1.00	0.19	0.59	0.30	0.51	1.00	0.52	0.53	2.42	1.03	1.00	0.74	0.71	1.38	1.49
IFNAR1	1.00	0.78	1.01	0.87	1.11	1.00	0.76	0.61	0.86	0.97	1.00	0.79	0.56	0.76	0.66
IFNAR2	1.00	1.26	1.72	0.90	1.20	1.00	1.05	1.33	0.90	0.75	1.00	0.80	1.06	0.70	0.94
IFNGR1	1.00	1.08	0.74	0.52	1.32	1.00	1.01	0.74	0.74	1.70	1.00	0.97	0.64	0.94	1.20
IL4R	1.00	1.36	1.41	1.23	1.22	1.00	0.86	0.87	0.78	1.19	1.00	0.86	1.31	1.29	1.07
IL12RB1	1.00	0.69	0.54	0.26	0.47	1.00	2.30	2.74	2.05	3.01	1.00	1.56	2.55	3.81	2.11
IL12RB2	1.00	0.00	1.47	1.36	0.62	1.00	0.37	1.99	1.10	1.11	1.00	3.30	4.49	5.57	0.00
IL13RA1	1.00	1.06	1.04	0.50	1.05	1.00	1.59	1.32	1.30	2.39	1.00	1.95	1.87	1.11	2.10
IL13RA2	1.00	0.00	0.00	33.6	14.14	1.00	1.73	1.78	5.79	1.69	1.00	1.02	1.14	3.04	1.95
IL15RA	1.00	1.23	3.00	1.02	1.03	1.00	3.37	7.94	0.79	0.88	1.00	3.21	8.89	1.12	0.87
IL17RA	1.00	1.56	0.90	2.73	1.60	1.00	0.40	2.01	2.17	1.27	1.00	0.78	0.80	0.46	0.29
IL17RC	1.00	1.31	1.61	0.71	1.35	1.00	1.44	1.66	1.13	1.25	1.00	1.60	1.15	1.39	1.44
STAT1	1.00	3.28	4.77	0.52	0.78	1.00	19.48	10.73	1.05	1.00	1.00	30.08	17.39	1.22	1.41
STAT2	1.00	1.89	1.94	0.53	0.68	1.00	5.78	3.25	0.92	1.06	1.00	8.96	4.96	1.15	1.10
STAT3	1.00	1.11	1.17	0.50	0.78	1.00	1.11	1.14	0.71	1.02	1.00	1.30	1.65	1.32	1.35
STAT4	1.00	1.13	0.53	0.07	1.12	1.00	1.20	1.03	0.76	1.00	1.00	0.75	0.52	1.26	2.61
STAT5A	1.00	1.17	0.86	0.22	0.54	1.00	1.65	4.90	0.70	1.88	1.00	3.97	2.91	0.89	1.23
STAT5B	1.00	0.79	0.69	0.38	0.63	1.00	2.61	2.26	1.52	2.08	1.00	1.37	2.51	2.59	1.48
STAT6	1.00	0.97	0.88	0.39	0.53	1.00	1.18	0.67	0.83	1.04	1.00	1.13	0.93	1.27	1.26
JAK1	1.00	1.06	1.07	1.55	1.40	1.00	1.22	1.54	1.13	1.21	1.00	1.22	0.94	0.85	1.02
JAK2	1.00	1.73	3.62	0.68	1.28	1.00	2.11	3.16	1.05	1.24	1.00	4.11	2.86	1.28	0.59
JAK3	1.00	1.00	0.74	0.53	0.68	1.00	2.01	2.31	0.83	1.63	1.00	1.76	2.72	2.09	1.36
TYK2	1.00	0.88	0.94	0.64	1.06	1.00	1.00	0.79	1.09	1.27	1.00	1.01	1.12	1.37	1.32
LCN2	1.00	1.13	0.96	0.93	2.73	1.00	0.76	0.61	0.58	1.72	1.00	1.39	1.09	1.02	2.40
FABP5	1.00	1.12	1.10	1.65	1.16	1.00	1.30	1.17	0.96	0.96	1.00	1.55	1.00	0.47	0.57
AQP3	1.00	0.89	0.86	0.47	0.70	1.00	0.85	0.85	0.72	0.85	1.00	1.01	0.64	1.26	0.97
SLC25A1	1.00	1.76	2.87	2.05	3.07	1.00	1.12	1.72	1.93	2.06	1.00	1.76	1.81	3.02	2.62
SOCS2	1.00	1.43	1.13	0.65	1.47	1.00	1.21	0.45	0.96	0.90	1.00	0.64	0.69	2.64	2.18
SOCS3	1.00	1.86	2.78	0.63	1.90	1.00	0.86	2.94	1.18	2.32	1.00	0.95	8.43	3.65	1.94
BCL6	1.00	0.77	0.88	0.80	1.09	1.00	0.76	1.68	0.84	0.80	1.00	0.99	0.88	1.02	0.66
CISH	1.00	1.34	1.23	9.76	1.34	1.00	0.39	0.81	4.72	0.94	1.00	0.81	1.23	8.07	1.05

## References

[R1] Bolger A.M., Lohse M., and Usadel B. (2014). Trimmomatic: a flexible trimmer for Illumina sequence data. Bioinformatics 30, 2114–2120. 24695404 10.1093/bioinformatics/btu170 PMC4103590

[R2] Brann (2020). Non-neuronal expression of SARS-CoV-2 entry genes in the olfactory system suggests mechanisms underlying COVID-19-associated anosmia. BioRxiv. 10.1126/sciadv.abc5801 PMC10715684 32937591

[R3] Cui Y., Hosui A., Sun R., Shen K., Gavrilova O., Chen W., Cam M.C., Gao B., Robinson G.W., and Hennighausen L. (2007). Loss of signal transducer and activator of transcription 5 leads to hepatosteatosis and impaired liver regeneration. Hepatology 46, 504–513. 17640041 10.1002/hep.21713

[R4] Heinz S., Benner C., Spann N., Bertolino E., Lin Y.C., Laslo P., Cheng J.X., Murre C., Singh H., and Glass C.K. (2010). Simple combinations of lineage-determining transcription factors prime cis-regulatory elements required for macrophage and B cell identities. Mol Cell 38, 576–589. 20513432 10.1016/j.molcel.2010.05.004 PMC2898526

[R5] Hennighausen L., and Robinson G.W. (2008). Interpretation of cytokine signaling through the transcription factors STAT5A and STAT5B. Genes Dev 22, 711–721. 18347089 10.1101/gad.1643908 PMC2394721

[R6] Hoffmann M., Kleine-Weber H., Schroeder S., Kruger N., Herrler T., Erichsen S., Schiergens T.S., Herrler G., Wu N.H., Nitsche A., (2020). SARS-CoV-2 Cell Entry Depends on ACE2 and TMPRSS2 and Is Blocked by a Clinically Proven Protease Inhibitor. Cell 181, 271–280 e278. 32142651 10.1016/j.cell.2020.02.052 PMC7102627

[R7] Imai Y., Kuba K., Rao S., Huan Y., Guo F., Guan B., Yang P., Sarao R., Wada T., Leong-Poi H., (2005). Angiotensin-converting enzyme 2 protects from severe acute lung failure. Nature 436, 112–116. 16001071 10.1038/nature03712 PMC7094998

[R8] Lamers M.M., Beumer J., van der Vaart J., Knoops K., Puschhof J., Breugem T.I., Ravelli R.B.G., Paul van Schayck J., Mykytyn A.Z., Duimel H.Q., (2020). SARS-CoV-2 productively infects human gut enterocytes. Science. 10.1126/science.abc1669 PMC7199907 32358202

[R9] Langmead B., Trapnell C., Pop M., and Salzberg S.L. (2009). Ultrafast and memory efficient alignment of short DNA sequences to the human genome. Genome Biol 10, R25. 19261174 10.1186/gb-2009-10-3-r25 PMC2690996

[R10] Liu X., Robinson G.W., Wagner K.U., Garrett L., Wynshaw-Boris A., and Hennighausen L. (1997). Stat5a is mandatory for adult mammary gland development and lactogenesis. Genes Dev 11, 179–186. 9009201 10.1101/gad.11.2.179

[R11] Love M.I., Huber W., and Anders S. (2014). Moderated estimation of fold change and dispersion for RNA-seq data with DESeq2. Genome Biol 15, 550. 25516281 10.1186/s13059-014-0550-8 PMC4302049

[R12] Lukassen S., Chua R.L., Trefzer T., Kahn N.C., Schneider M.A., Muley T., Winter H., Meister M., Veith C., Boots A.W., (2020). SARS-CoV-2 receptor ACE2 and TMPRSS2 are primarily expressed in bronchial transient secretory cells. EMBO J, e105114. 32246845 10.15252/embj.20105114 PMC7232010

[R13] Masella A.P., Lalansingh C.M., Sivasundaram P., Fraser M., Bristow R.G., and Boutros P.C. (2016). BAMQL: a query language for extracting reads from BAM files. BMC Bioinformatics 17, 305. 27516195 10.1186/s12859-016-1162-y PMC4982315

[R14] Qi F., Qian S., Zhang S., and Zhang Z. (2020). Single cell RNA sequencing of 13 human tissues identify cell types and receptors of human coronaviruses. Biochem Biophys Res Commun 526, 135–140. 32199615 10.1016/j.bbrc.2020.03.044 PMC7156119

[R15] Ramirez F., Ryan D.P., Gruning B., Bhardwaj V., Kilpert F., Richter A.S., Heyne S., Dundar F., and Manke T. (2016). deepTools2: a next generation web server for deep sequencing data analysis. Nucleic Acids Res 44, W160–165. 27079975 10.1093/nar/gkw257 PMC4987876

[R16] Shin H.Y., Willi M., HyunYoo K., Zeng X., Wang C., Metser G., and Hennighausen L. (2016). Hierarchy within the mammary STAT5-driven Wap super-enhancer. Nat Genet 48, 904–911. 27376239 10.1038/ng.3606 PMC4963296

[R17] Stark G.R., and Darnell J.E. Jr. (2012). The JAK-STAT pathway at twenty. Immunity 36, 503–514. 22520844 10.1016/j.immuni.2012.03.013 PMC3909993

[R18] Thorvaldsdottir H., Robinson J.T., and Mesirov J.P. (2013). Integrative Genomics Viewer (IGV): high-performance genomics data visualization and exploration. Brief Bioinform 14, 178–192. 22517427 10.1093/bib/bbs017 PMC3603213

[R19] Wu Y., Liu C., Dong L., Zhang C., Chen Y., Liu J., Zhang C., Duan C., Zhang H., Mol B.W., (2020). Coronavirus disease 2019 among pregnant Chinese women: Case series data on the safety of vaginal birth and breastfeeding. BJOG. 10.1111/1471-0528.16276 PMC7383704 32369656

[R20] Yuasa K., and Hijikata T. (2016). Distal regulatory element of the STAT1 gene potentially mediates positive feedback control of STAT1 expression. Genes Cells 21, 25–40. 26592235 10.1111/gtc.12316

[R21] Zhao Y. (2020). Single-cell RNA expression profiling of ACE2, the receptor of SARS-CoV-2. BioRxiv. 10.1164/rccm.202001-0179LE PMC7462411 32663409

[R22] Ziegler (2020). SARS-CoV-2 receptor ACE2 is an interferon-stimulated gene in human airway epithelial cells and is detected in specific cell subsets across tissues. Cell. 10.1016/j.cell.2020.04.035 PMC7252096 32413319

